# Cotton Gauze Impregnated With Chitosan Nanoparticle‐Loaded Recombinant Human Lactoferrin for Targeting Bacteria‐Infected Wounds: An In Vitro Study

**DOI:** 10.1155/ijm/9313676

**Published:** 2026-07-22

**Authors:** Aseel Mahmood Ibrahim Al-Mashahedah

**Affiliations:** ^1^ Department of Biology, College of Education, Al-Iraqia University, Baghdad, Iraq, aliraqia.edu.iq

**Keywords:** antibacterial agent, antibiofilm agent, chitosan, cotton gauze, nanocomposite, recombinant human lactoferrin

## Abstract

**Background:**

A bacterial infection during wound healing remains one of the most common complications in wound care. It not only impedes the wound‐healing process but can also result in chronic wounds, which contribute to increased morbidity and even mortality. Therefore, there is an urgent need to develop effective wound dressings to reduce wound infections.

**Methods:**

Chitosan nanoparticle‐loaded recombinant human lactoferrin (CHNP‐rhLF) was formulated by the ionotropic gelation method. Physicochemical characterizations of CHNP‐rhLF were assessed, including E.E%, L.C%, Yelid%, TEM, *ζ*‐potential, and drug release kinetics. To achieve modern wound‐dressing properties, cotton gauze samples were impregnated with 5%, 10%, and 15% (w/v) CHNP‐rhLF by dip, dry, and cure methods. The vertical wicking and swelling capacity of CHNP‐rhLF‐treated cotton gauze were then estimated. Antimicrobial and antibiofilm activities of CHNP‐rhLF‐treated cotton gauze were also evaluated against methicillin‐resistant *Staphylococcus aureus* (MRSA) and *Pseudomonas aeruginosa (P. aeruginosa)* using standard methods.

**Results:**

The results demonstrated successful encapsulation of rhLF within the chitosan matrix and subsequent drug release over 120 h. Posttreating cotton gauze with 5%, 10%, and 15% CHNP‐rhLF, the vertical wicking and swelling capacities improved. Vertical wicking values of CHNP‐rhLF‐treated cotton gauze at 10 min were 61 ± 1, 63 ± 1.2, and 66.4 ± 0.8 mm, respectively. Similarly, swelling capacity values reached 264.9% ± 0.9*%*, 271% ± 2.5*%*, and 285% ± 2*%*, respectively. Additionally, CHNP‐rhLF‐treated cotton gauze exhibited higher antibacterial efficiency against *P. aeruginosa* than MRSA in both planktonic and adherent phases, particularly at a concentration of 15%.

**Conclusion:**

Multifunctional properties of CHNP‐rhLF‐treated cotton gauze could make it a promising therapeutic approach for wound dressing.

## 1. Introduction

Human skin is the largest organ in the body and has a fundamental role in protecting and maintaining the body′s integrity [1]. However, it may be exposed to various external factors (such as diabetic foot ulcers and eczema), resulting in hard‐to‐heal (chronic) wounds [2]. Globally, approximately 1.5–2 million people have been reported to suffer from developing a wound annually in Europe alone [3]. Economically, French health insurance data for 2022 showed that wound dressings accounted for the second‐largest expenditure category at €732 million. Accordingly, wounds not only affect the patients′ quality of life scale but also constitute an economic burden on healthcare systems [4].

From a microbiological perspective, skin wounds supply a warm, moist, and nutritious environment favorable for colonization and proliferation of microbes [5, 6]. Invasive bacteria act to create a cytotoxic and harmful wound environment. Invasive bacteria can also cluster together to form biofilms, which are defined as microbial aggregates embedded in a self‐produced polymeric matrix of polysaccharides, proteins, and DNA. The biofilm is initially formed by planktonic bacteria, which reversibly attach to a wound surface. This is followed by the irreversibly binding of bacteria growing in biofilm to the surface and then multiplying to produce a slimy polymeric matrix around the microcolonies. The biofilm gradually grows in thickness and shows high resistance to antibiotics [7, 8]. In this aspect, several studies indicated that bacteria living in the biofilm have 10‐ to 1000‐fold higher antibiotic resistance than those living in a planktonic state. Therefore, proactive and appropriate wound management is essential to improve clinical outcomes [9–11].

In the biomedical field, cotton gauze is widely used as a wound dressing. Although this material′s biocompatibility and ability to absorb wound exudate represent an advantage, it also has some limitations. One of them is that cotton gauze can host pathogenic microorganisms in its structure, causing wound infections. In this aspect, many studies have focused on modifying the functional groups or antimicrobial agents of the cotton gauze polymer structure to prevent microbial invasion and improve wound healing [12].

Chitosan has been documented for its application in wound dressings [13–15]. Chitosan is a polysaccharide with dual functions: (i) antimicrobial and (ii) wound‐healing accelerator by activating immune cells and stimulating Type IV collagen synthesis. Chitosan has further properties such as biocompatibility and biodegradability, making it safe for use in medical applications [16].

Similarly, lactoferrin is an iron‐binding glycoprotein and is a member of the transferrin family. Lactoferrin has been proven to have a broad spectrum of antimicrobial activities. Recent studies have also revealed that lactoferrin can regulate the growth, migration, and differentiation of many cell types, including dermal fibroblasts. This in turn enhances wound contraction and accelerates the wound‐healing process [17, 18].

Inspired by this, we assumed that combining chitosan and lactoferrin in a nanosystem could constitute a therapeutic strategy for wound care. Therefore, cotton gauze treated with chitosan nanoparticle‐loaded lactoferrin was designed. This study is aimed at formulate bioactive cotton gauzes with high absorbency and antimicrobial activity while maintaining the inherent textile characteristics of cotton dressings, thereby creating an environment that minimizes infection‐related complications and accelerates recovery.

## 2. Materials and Methods

### 2.1. Synthesis of Chitosan Nanoparticle‐Loaded Recombinant Human Lactoferrin (CHNP‐rhLF)

The CHNP‐rhLF was synthesized using the ionotropic gelation method [19]. A 0.05% w/v solution of chitosan (120 kDa, DD ≥ 90*%*) (Glentham Life Sciences, UK) was prepared by dissolving chitosan powder in an acetic acid solution with a pH of 5.0. Ten milliliters of recombinant human lactoferrin (rhLF) (Sino Biological, United States) aqueous solution (0.1% w/v) was added dropwise to 100 mL of prepared chitosan solution. The mixture was then stirred at 200 rpm for 24 h at 4°C. Afterward, rhLF‐loaded CHNPs were formulated by dropping 20 mL of sodium tripolyphosphate (STPP) (HiMedia Laboratories Pvt, India) aqueous solution (0.1% w/v) to 110 mL of the mixture. Stirring was continued under the same conditions for 1 h to complete the crosslinking. The mixture was then subjected to ultrasonic power of 45 W for 5 min at 4°C. The nanoparticle pellet was obtained after centrifugation at 4000 rpm for 30 min at 4°C. Meanwhile, the suspension was collected to determine the free protein content. The pellet was lyophilized and finally stored at −20°C until further use.

The free protein content was detected using the Bradford spectrophotometric assay at OD_595_nm. The unknown amount of free protein was determined according to the bovine serum albumin (BSA) standard curve. The encapsulation efficiency (E.E%), loading capacity (L.C%), and yield (Yield%) of CHNP‐rhLF were then evaluated using the following equations:

E.E%=Total protein mg−Free protein in supernatant mgTotal protein mg×100.

L.C%=Total protein mg−Free protein in supernatant mgNanoparticle weight mg×100.

Yield%=Nanoparticle weight mgTotal solid weight of polymer,protein,and STPP mg×100.

### 2.2. Transmission Electron Microscope (TEM)

The particle shape and size of CHNP‐rhLF were identified using a TEM (JEM JEOL 2100). CHNP‐rhLF samples were prepared by drop‐coating on copper grids and dried overnight. The samples were subjected to TEM analysis at an accelerating voltage of 200 kV. TEM images obtained were then used to detect the particle size of CHNP‐rhLF using the ImageJ software [20].

### 2.3. Zeta‐Potential (*ζ*)

The *ζ*‐potential of CHNP‐rhLF was evaluated using the Malvern Zetasizer Nano‐ZS (ATA Scientific) instrument based on electrophoretic mobility measurements. The CHNP‐rhLF samples were prepared at a concentration of 0.1% and then serially diluted to a suitable dilution. The *ζ*‐potential of CHNP‐rhLF samples was measured using the BI‐SCP cell (ATA Scientific) at 25°C, and the obtained data were analyzed [21].

### 2.4. Drug Release Kinetics

To investigate the success of the encapsulation process of rhLF protein inside the chitosan matrix and then the release of protein from encapsulated nanoparticles, the kinetics of drug release were examined. The simulated wound fluid (sodium chloride [584.4 mg], sodium hydrogen carbonate [336 mg], potassium chloride [29.82 mg], calcium chloride [27.75 mg], bovine albumin [3300 mg], dissolved in deionized water [100 mL]) was made at a pH of 8.0 [22, 23]. The samples of CHNP‐rhLF were suspended by adding 5 mg of CHNP‐rhLF to 1 mL of simulated wound fluid and incubating at 37°C under steady rotation at 70 rpm. Bradford assay was applied to evaluate the release rate of protein at regular periods (0, 0.5, 1, 2, 4, 6, 8, 10, 12, 24, 48, 72, 96, and 120 h) with a spectrophotometer at OD_595_nm. The quantity of protein release was estimated based on the BSA standard curve [24].

### 2.5. Preparation of Treated Cotton Gauzes With CHNP‐rhLF

Traditional cotton gauzes were purchased from a local supplier. The cotton gauzes were immersed in the solution of CHNP‐rhLF at different concentrations (5%, 10%, 15% w/v) for 2 min and then left to dry. To improve the coverage of cotton gauzes, they were dipped in a sodium alginate (Na–AG) (Sigma–Aldrich) solution (1% w/v) for 2 min. Finally, the treated wound dressings were placed on a glass support to dry [25].

The kinetics of drug release from the treated cotton gauze with CHNP‐rhLF were investigated. A 2 × 2 cm piece of treated cotton gauze was immersed in 5 mL of simulated wound fluid and gently rocked. The Bradford assay was performed to estimate the time‐dependent release rate of rhLF protein from CHNP‐rhLF.

### 2.6. Vertical Wicking Test

A vertical wicking test was carried out to measure the water transport rate. Treated cotton gauzes with CHNP‐rhLF were prepared at 17 × 2.5 cm diameter. Three millimeters of one end of the treated cotton gauze strip were immersed in distilled water, to which 1% Prussian blue staining was added to track the movement of water. The height of water transported along the strip was quantified at 1, 5, and 10 min [26].

### 2.7. Swelling Capacity Degree

The swelling capacity degree, percent of treated cotton gauzes with CHNP‐rhLF, was determined using the gravimetric method. This was conducted by preparing pieces of treated cotton gauze with a diameter of 5 × 5 cm. Each dry‐treated cotton gauze was accurately weighed and then immersed in 30 mL of distilled water for 30 min at room temperature. After incubation, each wet‐treated cotton gauze was weighed after removing excess water from the gauze surface by gently blotting with filter paper. The swelling capacity degree percent of treated cotton gauzes with CHNP‐rhLF was evaluated via the following equation [27]:

The Swelling Capacity Degree%=Wwet−WdryWdry×100,

wherein W_dry_is the dry weight of gauzes and W_wet_ is the wet weight of gauzes.

### 2.8. Bacterial Clinical Isolation and Culture

Isolated methicillin‐resistant *Staphylococcus aureus* (MRSA) and *Pseudomonas aeruginosa* from patients with infected wounds were supplied by the laboratories of Al Yarmouk Teaching Hospital and Al Karama Teaching Hospital, Baghdad, Iraq. The isolation and examination of the clinical bacterial samples were carried out under the supervision of specialist physicians. The bacterial samples underwent an exhaustive examination that included sample culture and bacteriological assays for differential diagnosis and determination of the specific pathogen type. Clinical bacterial isolates were routinely preserved on nutrient agar slants at 4°C for experimental use.

### 2.9. Antimicrobial Activity Studies

Antimicrobial activities of treated cotton gauzes with CHNP‐rhLF were investigated against MRSA and *P. aeruginosa* using the following standard methods:

#### 2.9.1. Disc Diffusion Method

The disc diffusion method was performed using the Kirby‐Bauer disc diffusion method [28]. Initially, treated cotton gauzes with CHNP‐rhLF (5%, 10%, and 15%  w/v) were cut as discs with a 1.5 cm diameter and sterilized by UV for 30 min. Untreated cotton gauze discs were used as a negative control. Mueller–Hinton agar plates were prepared, sterilized, and allowed to solidify at room temperature. The plates were then inoculated with 1.5 × 10^8^ colony‐forming units/mL (0.5 McFarland) bacterial suspensions prepared from MRSA and *P. aeruginosa* using a sterile swab. Afterward, the discs of untreated and treated cotton gauzes with CHNP‐rhLF were placed on the inoculated plates. The inoculated plates were then incubated for 24 h at 37°C. Finally, the zone diameters of inhibition were measured and recorded.

#### 2.9.2. The Colony‐Forming Count Method

The colony‐forming count method was used to assess the efficacy of treated cotton gauzes with CHNP‐rhLF against the growth of MRSA and *P. aeruginosa*. This method was carried out in both cases: (i) planktonic cultures (free‐floating bacterial cells) and (ii) gauze surface‐attached (adherent bacterial cells). The sterilized discs (1.5 cm) of the cotton gauze treated with 5%, 10%, and 15% CHNP‐rhLF were used, whereas the untreated cotton gauze was utilized as a control. These discs were then placed in a 24‐well plate and immersed in 2 mL sterile Mueller–Hinton broth. Afterward, the wells were inoculated with 20 *μ*L of 1 × 10^5^ CFU/mL of MRSA or *P. aeruginosa* and incubated in a shaking incubator for 24 h at 37°C. After incubation, planktonic suspensions were serially diluted. One hundred microliters of each was then drawn and spread onto the Mueller–Hinton agar plate. The plates were incubated for 24 h at 37°C. The viable bacterial colonies were counted, and the CFU/mL values were determined as planktonic bacteria. In the same context, the incubated gauze discs were used to assess surface‐adherent bacteria on the gauze. The discs were gently washed with sterile normal saline and then placed in sterile Eppendorf tubes containing 1 mL of sterile normal saline. Eppendorf tubes were vigorously vortexed to detach the attached bacterial cells. The suspensions were diluted and inoculated on Mueller–Hinton agar plates. CFU/mL values of MRSA and *P. aeruginosa* were detected. The data obtained were eventually expressed as log base [25, 29].

### 2.10. Statistical Analysis

Statistical analysis was performed using GraphPad Prism (GraphPad Software, Inc., San Diego, California, United States). All data were represented as mean ± SEM. Statistically significant differences were determined using Student′s *t*‐test for two groups, and one‐way analysis of variance (ANOVA) was used for more than two groups. The level of statistical significance was accepted at *p* < 0.05, where ∗: *p* < 0.05, ∗∗: *p* < 0.01, ∗∗∗: *p* < 0.001, ∗∗∗∗: *p* < 0.0001. All experiments were conducted in triplicate, and the representative data were presented.

## 3. Results

Chitosan nanoparticles loaded with rhLF were synthesized using the ionic gelation method. The E.E%, L.C%, Yield%, and size of the nanoparticles are important parameters considered in medical applications.

**Figure 1:**
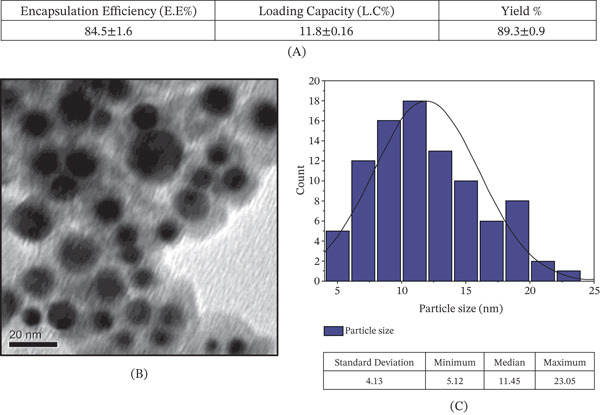
Characterization of chitosan nanoparticles loaded with rhLF. (A) The E.E%, L.C%, and Yield% values of CHNP‐rhLF. (B) The TEM image of CHNP‐rhLF. (C) The diagram of CHNP‐rhLF size distribution.

The results showed that the average E.E% of CHNP‐rhLF was 84.5*%* ± 1.6*%*, L.C% was 11.8*%* ± 0.16*%*, and Yield% was 89.3% ± 0.9% (Figure 1A). In the same context, the TEM analysis was performed to investigate the morphology and size distribution of CHNP‐rhLF. TEM images displayed that CHNP‐rhLF particles had almost spherical shapes (Figure 1B), with a mean size of 11.45 ± 4.13 nm (Figure 1C).

**Figure 2:**
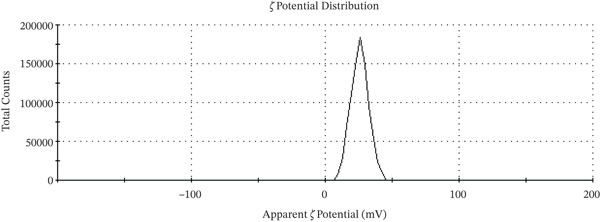
The *ζ*‐potential distribution of CHNP‐rhLF. The graph shows a positive *ζ*‐potential value of CHNP‐rhLF.

Based on electrophoretic mobility measurements, the *ζ*‐potential distribution outcomes demonstrated that the CHNP‐rhLF had a positive charge (25.7 ± 6.5 mV), as shown in Figure 2.

**Figure 3:**
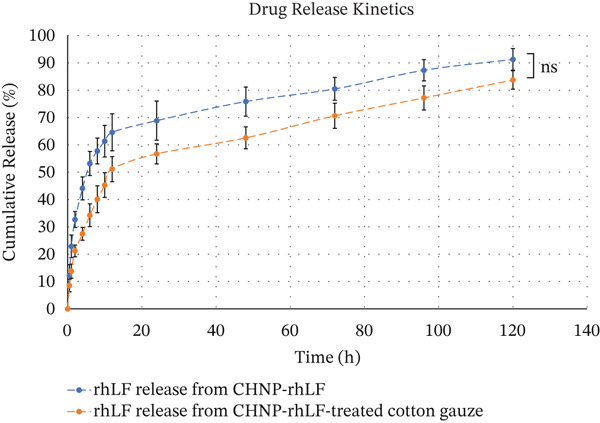
The release kinetics of rhLF from CHNP‐rhLF and CHNP‐rhLF‐treated cotton gauze. The release kinetics of rhLF from CHNP‐rhLF and CHNP‐rhLF‐treated cotton gauze were determined by a spectrophotometer at OD_595_nm. The protein release profiles were examined in the simulated wound fluid at 37°C and pH 8.0 within 0–120 h. The rhLF release data from CHNP‐rhLF and CHNP‐rhLF‐treated cotton gauze showed biphasic release kinetics with sustained release for more than 120 h.

The kinetics of drug release were also tracked. The results showed that the release kinetics of rhLF from CHNP‐rhLF and CHNP‐rhLF‐treated cotton gauze were biphasic, as illustrated in Figure 3. The initial burst release of rhLF from CHNP‐rhLF within 12 h was 64.5*%* ± 6.7*%* and 51.1*%* ± 4.6*%* from CHNP‐rhLF‐treated cotton gauze. This was followed by a slow and sustained release. At 120 h, the total release rate of rhLF from CHNP‐rhLF and CHNP‐rhLF‐treated cotton gauze was 91.3*%* ± 3.9*%* and 83.7*%* ± 3.4*%*, respectively, with no statistically significant differences between them, *p* = 0.22.

**Figure 4:**
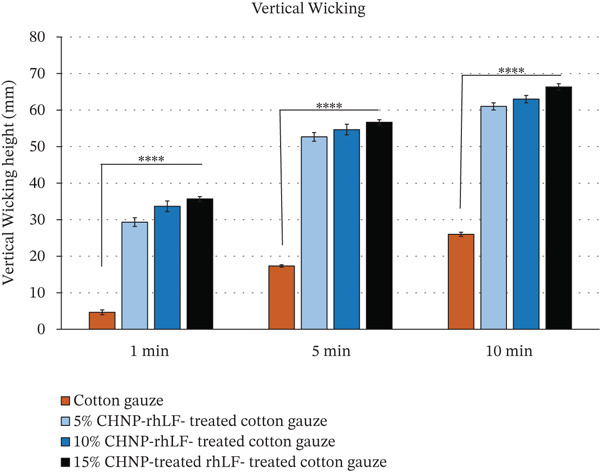
Vertical wicking behaviour of CHNP‐rhLF‐treated cotton gauze and untreated cotton gauze. The graph displays that the vertical wicking ability of 5%, 10%, and 15% CHNP‐rhLF‐treated cotton gauze was higher than that of untreated cotton gauze at different time points (1, 5, and 10 min).

The vertical wicking ability of CHNP‐rhLF‐treated and untreated cotton gauze samples was measured. The outcomes revealed that the vertical wicking ability of treated and untreated cotton gauze increased over time. In the first minute, it was observed that the vertical wicking ability of 5%, 10%, and 15% CHNP‐rhLF‐treated cotton gauze markedly increased (*p* < 0.0001) by 29.3 ± 1.2, 33.7 ± 1.5, and 35.6 ± 0.6 mm, respectively, compared to untreated cotton gauze 4.7 mm ± 0.7. The vertical wicking ability of CHNP‐rhLF‐treated cotton gauze was examined after 5 min, and a significant increase (*p* < 0.0001) was observed for all concentrations, reaching 52.7 ± 1.2 mm at 5%, 54.6 ± 1.3 mm at 10%, and 54.4 ± 1 mm at 15% compared with 17.3 ± 0.3 mm for untreated cotton gauze. Within 10 min, the results showed that the vertical wicking ability of 5%, 10%, and 15% CHNP‐rhLF‐treated cotton gauze reached 61 ± 1 mm, 63 ± 1.2 mm, and 66.4 ± 0.8 mm (*p* < 0.0001), respectively. In contrast, it reached 26 ± 0.5 mm for untreated cotton gauze (Figure 4).

**Figure 5:**
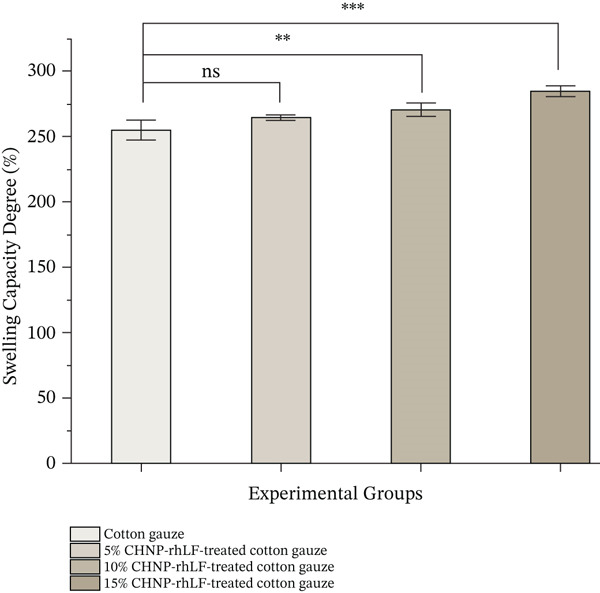
Swelling capacity of CHNP‐rhLF‐treated cotton gauze and untreated cotton gauze. The chart reveals that the swelling capacity value of cotton gauze was increased with the increase of CHNP‐rhLF concentration.

As expected, the untreated cotton gauze displayed a swelling capacity of 255.3*%* ± 3.8*%*. The swelling capacity was slightly raised (*p* = 0.07) to 264.9*%* ± 0.9*%* at 5% of CHNP‐rhLF‐treated cotton gauze, whereas the swelling value was markedly increased at 10% and 15% of CHNP‐rhLF‐treated cotton gauze, to be 271*%* ± 2.5*%* (*p* < 0.01) and 285*%* ± 2*%* (*p* < 0.001), respectively (Figure 5).

**Figure 6:**
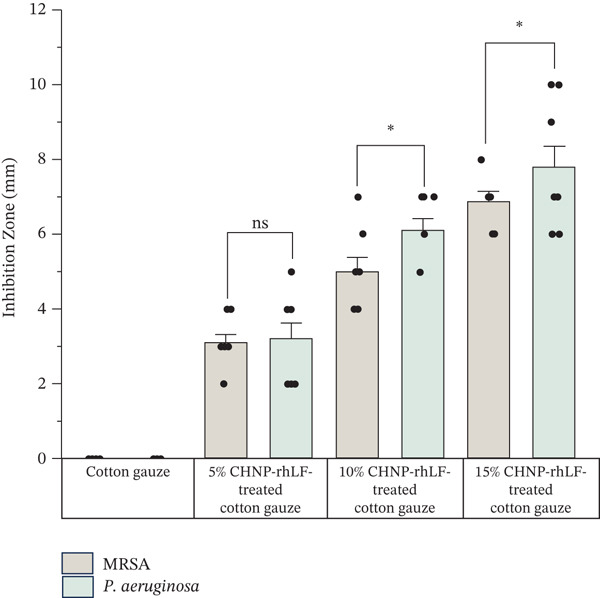
Evaluation of the antibacterial effect of CHNP‐rhLF‐treated cotton gauze by the disc diffusion method. The cotton gauze treated with 5%, 10%, and 15% CHNP‐rhLF showed antibacterial activity against MRSA and *P. aeruginosa*, with a notable effect on *P. aeruginosa*.

The antimicrobial activity of the cotton gauze treated with 5%, 10%, and 15% CHNP‐rhLF against MRSA and *P. aeruginosa* was assessed. As represented in Figure 6, the disc diffusion assay results showed that the 5% CHNP‐rhLF‐treated cotton gauze exhibited an inhibition zone of 3.1 ± 0.3 mm for MRSA and 3.2 ± 0.2 mm for *P. aeruginosa*, with no significant difference between them (*p* = 0.64). It was also observed that the efficiency of CHNP‐rhLF‐treated cotton gauze was upgraded with the increase of CHNP‐rhLF concentration. Where its effect was higher (*p* < 0.05) against *P. aeruginosa* compared to MRSA. For MRSA, the cotton gauze treated with 10% and 15% CHNP‐rhLF recorded inhibition zones of 5 ± 0.7 mm and 6.8 ± 0.1 mm, respectively. Whereas the inhibition zones of *P. aeruginosa* were 6.1 ± 0.4 mm for 10% CHNP‐rhLF and 7.8 ± 0.6 mm for 15% CHNP‐rhLF. No inhibition zone was noted with the untreated cotton gauze.

**Figure 7:**
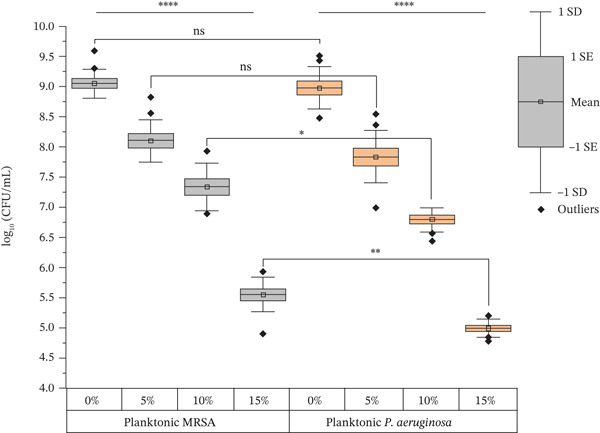
Inhibition of bacterial growth in the planktonic phase. Post‐24‐h incubation for the planktonic of MRSA or *P. aeruginosa* with the cotton gauze treated with 5%, 10%, and 15% CHNP‐rhLF, the bacterial growth was evaluated. A significant inhibition of bacterial growth was observed, particularly in *P. aeruginosa*.

The growth of MRSA and *P. aeruginosa* in plankton, containing cotton gauze treated with 5%, 10%, and 15% CHNP‐rhLF and untreated cotton gauze, was examined. The results showed a reduction in bacterial growth in the presence of CHNP‐rhLF‐treated cotton gauze compared to untreated cotton gauze (Figure 7). For MRSA, the CFU was 9 ± 0.2 log_10_ for untreated cotton gauze. This value was markedly decreased (*p* < 0.0001) to 8.1 ± 0.3 log_10_ CFU/mL at 5% CHNP‐rhLF, 7.3 ± 0.4 log_10_ CFU/mL at 10% CHNP‐rhLF, and 5.6 ± 0.2 log_10_ CFU/mL at 15% CHNP‐rhLF. Similarly, the CFU for *P. aeruginosa* was significantly reduced (*p* < 0.0001) when treated with 5%, 10%, and 15% of CHNP‐rhLF to 7.8 ± 0.4, 6.7 ± 0.3, and 5 ± 0.1 log_10_, respectively, compared with untreated cotton gauze 8.9 ± 0.3 log_10_ CFU/mL. The results also revealed that cotton gauze treated with 10% had a notable influence (*p* < 0.05) on the growth of *P. aeruginosa* than MRSA. This effect was further increased (*p* < 0.01) when treated with 15% CHNP‐rhLF.

**Figure 8:**
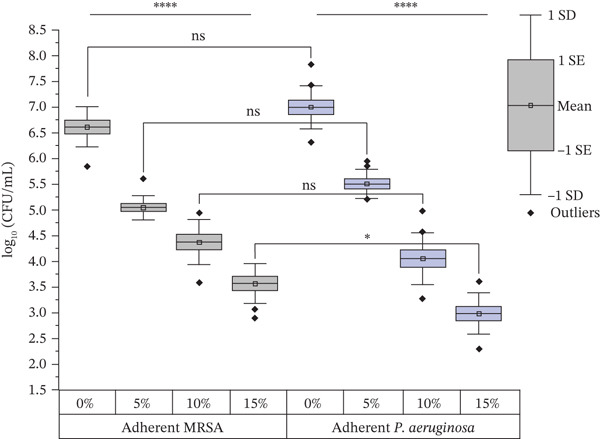
Inhibiting adherent bacterial cells on the cotton gauze surface. The cotton gauze treated with 5%, 10%, and 15% CHNP‐rhLF was able to inhibit adherent MRSA and *P. aeruginosa* and limit their biofilm formation.

The current study also addressed the efficiency of CHNP‐rhLF‐treated cotton gauze in inhibiting the process of bacterial cell adhesion to the cotton gauze surface, which is the first step in biofilm formation. Untreated cotton gauze samples demonstrated that the CFU of adherent MRSA was 6.6 ± 0.3 log_10_ and adherent *P. aeruginosa* was 7 ± 0.4 log_10_. This was significantly decreased (*p* < 0.0001) when cotton gauze was treated with 5%, 10%, and 15% CHNP‐rhLF, reaching 5 ± 0.2, 4.3 ± 0.4, and 3.5 ± 0.4 log_10_ CFU/mL of adherent MRSA and 5.5 ± 0.3, 4 ± 0.5, and 2.9 ± 0.3 log_10_ CFU/mL of adherent *P. aeruginosa* (Figure 8).

## 4. Discussion

The design of antibacterial dressings is still crucial to overcoming the challenges of wound infection and promoting the healing process. Contemporary theories of wound dressings suggest ideal parameters, which are (i) maintaining a balance between exudate absorption and surface moisture while allowing gas exchange for sufficient oxygen and (ii) acting against microorganisms [30–32]. In this aspect, cotton gauze fabrics (old‐style classical wound dressing) have many features, including low cost, easy manufacturing, and high permeability and absorption [33]. Moreover, the molecular structure, polar functional groups, and large active surface area of cellulose fibers can introduce an ideal material for designing bioactive and biocompatible multifunctional materials [27]. However, the moisture retention and organic composition of cotton gauze can support bacterial colonization of the wound [31]. Accordingly, conventional cotton gauze treated with chitosan nanoparticles loaded with rhLF was used in this study in order to create an environment that prevents bacterial invasion and accelerates wound healing.

The physicochemical characterizations of the synthesized CHNP‐rhLF demonstrated the successful encapsulation process of lactoferrin into the chitosan polymer matrix. The TEM images also revealed the predominant spherical shape of CHNP‐rhLF with an average size in the nanoscale range. This is a desirable outcome, as a high surface area aspect ratio of nanoparticles can bind large amounts of protein, thus enhancing their bioavailability [34]. In the same context, the positive *ζ*‐potential value of CHNP‐rhLF indicates that nanoparticles are stable. This may be due to high electrostatic repulsion between the particles [35].

After verifying the possibility of incorporating rhLF into the chitosan matrix, the release pattern of rhLF from chitosan nanoparticles before and after their placement in the bandage was studied. This study also investigated whether covering the cotton gauze with Na–AG hindered the drug release. The findings revealed that the release of rhLF from CHNPs before and after their placement in the bandage was biphasic, which includes an initial burst phase followed by a sustained release phase over 120 h. This fits with the suggested release mechanisms, which are carried out in various stages are as follows: (i) the drug desorption from the particle surface, (ii) drug diffusion and reabsorption by the pores of the polymer network, and (iii) the deterioration of the polymeric network and the drug release into the external medium [36]. The biphasic release characteristics of the rhLF‐loaded chitosan nanoparticles could be a sign of rhLF availability in the wound site by ensuring a constant rhLF delivery rate for at least 120 h after the application. Additionally, the drug retention within the chitosan matrix for long periods can screen the surrounding tissues from any negative side effects related to excessive drug concentrations [37].

The findings further demonstrated that CHNP‐rhLF‐treated cotton gauze could maintain a higher moisture environment than untreated cotton gauze. These findings are consistent with what [26, 27] found, which indicated that chitosan nanoparticles were able to enhance the absorption capacity of cotton gauze. The existence of hydroxyl and carboxyl functional groups (OH/COOH) within cotton gauze can exhibit hydrophilic characteristics capable of absorbing water. These hydrophilic attributes are significantly enhanced when the chitosan nanoparticles are applied to the surfaces of the cotton gauze, which results in the formation of additional hydrophilic groups, specifically NH_2_ and OH groups [38]. Furthermore, chitosan nanoparticles possess the ability to occupy the voids within cotton gauze formed by the intersections of warp and weft fibers. Because of the inherent channel‐like structure of chitosan, the capillary number of cotton gauze increases in both the warp and weft directions. Consequently, this advancement facilitates the vertical wicking and subsequent absorption of water [26]. In this regard, several studies demonstrated that increasing the swelling capacity and maintaining a moist environment for the cotton gauze play an important role in the wound healing process. This is due to (i) increasing the absorption of wound exudates and (ii) preventing the formation of scabs or scars, which can form a mechanical barrier that hinders the migration of epidermal cells and forces them to move deeper, causing delayed healing [39, 40].

The most important aspect of this study was to determine whether the formulated dressings could inhibit bacterial growth that causes wound infections. Therefore, these dressings were applied to *P. aeruginosa* and MRSA isolated from infected wounds. Both bacteria belong to the ESKAPE pathogen group, which is known for its high multidrug resistance (MDR) and extreme virulence [41].

The data revealed that the developed dressings (CHNP‐rhLF‐treated cotton gauze) had antimicrobial activity against both planktonic forms, MRSA and *P. aeruginosa*. This could be attributed to the antimicrobial properties of chitosan nanoparticles, such as their large surface area and small size. The relatively large surface area of nanoparticles provides more cationic sites than free chitosan. The cationic sites, in turn, allow the nanoparticles to interact tightly with the bacterial surface charge. The resulting ionic interaction leads to bacterial membrane depolarization, membrane disruption, and ultimately more leakage of intracellular substances outside bacteria [42, 43]. Additionally, the small size of nanoparticles allows them to penetrate bacterial membranes and damage organelles, leading to bacterial death [44]. In this regard, a study indicated that chitosan nanoparticles had a significant bacteriostatic effect of approximately 90% against *S. aureus* and *P. aeruginosa*, which cause skin infections; this aligns with our findings [45].

Similarly, formulated dressings demonstrated remarkable antiadhesion effects against MRSA and *P. aeruginosa* on their surface, thus preventing biofilm formation. In line with a study showing that chitosan nanoparticles, in addition to their bacteriostatic effect on Staphylococcal species, including MRSA, were also able to inhibit their adhesion to polystyrene treated with rabbit plasma [46]. Our results are also consistent with another study that found chitosan nanoparticles can inhibit *P. aeruginosa*. Adhesion on the polystyrene surface and limit biofilm formation [47]. Chitosan nanoparticles can impede bacterial adhesion by interfering with the initial steps of the adherence process. This may occur via multiple mechanisms, including (i) electrostatic interaction with bacterial surfaces, (ii) disturbing membrane proteins implicated in adhesion, (iii) blocking adhesion sites by forming a thin coating of chitosan nanoparticles on surfaces and occupying bacterial binding sites, (iv) inhibition of biofilm matrix production through suppressing extracellular polymeric substance (EPS) production and preventing stable biofilm maturation, and (vi) surface hydrophobicity modification, which in turn reduces bacterial affinity for the surface and colonization [48–50].

Owing to rhLF properties, it was encapsulated within a chitosan matrix to promote antibacterial and anticolonization activity. Lactoferrin is an iron‐chelating protein that can disrupt iron regulation of bacterial biochemical and metabolic functions, which are fundamental for bacterial growth and development. Lactoferrin can also hinder the first step of biofilm formation by preventing lectin‐dependent bacterial motility or adhesion to surfaces [51]. Lactoferrin can also nullify the biofilms′ scavenging system for collecting essential minerals and nutrients, which may diffuse via bacterial biofilms [52].

Accordingly, combining the properties of cotton gauze and rhLF in a chitosan eco‐friendly nanosystem, along with providing high bioavailability and a sustained bacteriotoxic effect over 120 h, could play a bacteriostatic role and reduce the invasion and colonization of the wound by MRSA and *P. aeruginosa*. This approach, aligned with next‐generation wound care trends, can inspire the development of sustainable medical innovations to accelerate the recovery of bacteria‐infected wounds.

## 5. Conclusion

With the increasing demands of wound care management, there exists a necessity for multifunctional wound dressings. To prevent the risk of infection and mitigate patient suffering, the antibacterial efficacy of wound dressing against a wide spectrum of bacterial strains, along with their wound‐healing properties, is among the most important characteristics to consider. Accordingly, cotton gauze treated with chitosan nanoparticle‐loaded rhLF was engineered in the current investigation. The outcomes revealed that CHNP‐rhLF‐treated cotton gauze had (i) a high fluid absorption, (ii) prolonged drug release over 120 h, (iii) antibacterial activity, and (iv) antibiofilm formulation. Hence, the implementation of a wound management strategy that integrates multifaceted approaches may demonstrate considerable promise, particularly in the context of severe wounds, serving as an efficacious system for infection prevention and wound care.

## Funding

No funding was received for this manuscript.

## Ethics Statement

No ethical approval is required. No identifiable personal information was collected or published. All bacterial samples were provided by the laboratories of Al Yarmouk Teaching Hospital and Al Karama Teaching Hospital, Baghdad, Iraq, without access to the patient data. All experiments adhered to the institutional biosafety guidelines and approvals (Iraqi Ministry of Health / Baghdad Health Directorate / Al‐Karkh, No: 50722 in June 2025) and to the relevant international standards for the use of bacteria.

## Conflicts of Interest

The author declares no conflicts of interest.

## Data Availability

The data that support the findings of this study are available from the corresponding author upon reasonable request
